# Cytokine Imprint in Preeclampsia

**DOI:** 10.3389/fimmu.2021.667841

**Published:** 2021-06-23

**Authors:** Katarzyna Stefańska, Maciej Zieliński, Martyna Jankowiak, Dorota Zamkowska, Justyna Sakowska, Przemysław Adamski, Joanna Jassem-Bobowicz, Karolina Piekarska, Katarzyna Leszczyńska, Renata Świątkowska-Stodulska, Sebastian Kwiatkowski, Krzysztof Preis, Piotr Trzonkowski, Natalia Marek-Trzonkowska

**Affiliations:** ^1^ Department of Obstetrics, Medical University of Gdańsk, Gdańsk, Poland; ^2^ Department of Medical Immunology, Medical University of Gdańsk, Gdańsk, Poland; ^3^ Department of Neonatology, Medical University of Gdańsk, Gdańsk, Poland; ^4^ Department of Endocrinology and Internal Medicine, Medical University of Gdańsk, Gdańsk, Poland; ^5^ Department of Obstetrics and Gynecology, Pomeranian Medical University of Szczecin, Szczecin, Poland; ^6^ International Centre for Cancer Vaccine Science Cancer Immunology Group, University of Gdansk, Gdańsk, Poland; ^7^ Laboratory of Immunoregulation and Cellular Therapies, Department of Family Medicine, Medical University of Gdańsk, Gdańsk, Poland

**Keywords:** preeclampsia, gestational hypertension, cytokine, inflammatory response, growth factors

## Abstract

The hallmark of preeclampsia (PE) is a shift toward persistent inflammatory response, accompanied by endothelial dysfunction. The driving forces in PE are proinflammatory cytokine and growth factors, in parallel with reduced functionality of anti-inflammatory effectors, like regulatory T cells are observed. Unfortunately, no conclusive mechanism underlying preeclampsia has been identified. For this reason, research on preeclampsia is needed to provide a state of the art understanding of the pathophysiology, identification of new diagnostics tools and the development of targeted therapies. The 68 patients were divided into three groups: gestational hypertension (GH) group (n = 19) and PE group (n = 28) and a control group (n = 21). We have tested a set of 53 cytokines, chemokines and growth factors in preeclampsia and gestational hypertension, and then compared them with normal pregnancies. Using a diagnostic test assessment characteristic parameters (IL-22, MDC/CCL22, IL-2/IL-4 ratio) have been identified and cut-off values have been proposed to diagnose preeclampsia. All parameters had high negative or positive predictive values, above 80%. In conclusion, we have proposed a potential set of immune parameters to diagnose preeclampsia.

## Introduction

Preeclampsia (PE) is a common complication during pregnancy, affecting 2–5% of pregnant women. What’s more, PE is one of the leading causes of perinatal morbidity worldwide, increasing the risk of mortality up to 15% due to obstetric complications. While in fetus PE is the primary cause of premature births and fetal growth restriction (FGR), the mother may develop eclampsia, renal/liver failure. Typically symptomatic women, previously normotensive, are diagnosed with hypertension over 140/90 mmHg and some of the following: proteinuria over 300 mg/24 h, maternal organ dysfunction, or uteroplacental dysfunction after the 20th week of gestation (1–5).

Although progress in unveiling the pathogenesis of preeclampsia has been made, the major mechanism, probably multifactorial, is still unknown. But one of the more intensively studied mechanisms is feto-maternal immunity. There is growing evidence that the progression of PE is associated with an imbalance between pro-and anti-inflammatory factors leading to a systemic response with the involvement of the vascular endothelium (6, 7). In the non-complicated pregnancy, immune cells such as natural killer cells, dendritic cells, and T regulatory lymphocytes located in the decidua, maintain immunotolerance toward spiral artery remodeling and emerging fetal trophoblast. Collectively, this local microenvironment plays a role in the maternal immune tolerance towards the fetal allograft, and abnormal placentation can lead to increased placental shedding, exaggerated systemic inflammation and subsequent endothelial dysfunction, the key characteristics of preeclampsia (8, 9).

A recent study on cytokine milieu in PE indicated several proteins, mainly cytokines and growth factors, that drive either inflammation or angiogenesis. This was primarily soluble endoglin (sEng) and soluble fms-like tyrosine kinase-1 (sFLT1) that acts similarly to scavengers, reducing circulating VEGF and PIGF. This severely affects angiogenic balance and endothelial function. In parallel, inflammation is forced by proinflammatory cytokines [IL-1β, TNF-α (tumor necrosis factor α), IL-6, IL-8, IL-1β and IL-18] secreted mainly by maternal immune cells (10–14). Also, trophoblast itself can maintain inflammation due to the production of interleukins [IL-1β, IL-2, IL-4, IL-6, IL-8, IL-12, TGFβ1 (transforming growth factor β1) and TNF-α], chemokines (MCP-1—monocyte chemoattractant protein-1), and an adhesion molecule [ICAM-1 (intercellular adhesion molecules-1), VCAM-1 (vascular cell adhesion molecule-1)] (11, 15–17).

Although to diagnose preeclampsia, hypertension and proteinuria are adequate, there is a great need for additional laboratory biomarkers, to predict, diagnose, or risk-stratification. Currently, there are relatively few markers used to screen for PE, essentially sFlt-1, PIGF, and PAPP-A (18, 19). There are protentional drawbacks of PE screening with the use of multiparametric algorithms. There are no defined testing time-points, lack of strict cut-offs when combined with ultrasonography for a uniform algorithm. Another was utilization of markers in the general gestational women population, or data comparison with the specific population from which they were taken (20). What’s more, new markers should point to PE before the symptoms of hypertension occur, as this pathology may affect both mother and fetus (21). Another important issue is to discriminate preeclampsia from gestational hypertension (GH), which has a better prognosis. To answer these questions, we made a study of several cytokines and growth factors in gestational hypertension and preeclamptic women. The results were then matched with the uncomplicated pregnancies, and, based on ROC analysis, the cut-off was set and sensitivity and specificity were calculated.

## Material and Methods

### Study Design

In this study, 68 women were enrolled from all hospitalized patients between April 2015 and December 2019 at the Department of Obstetrics, Medical University of Gdansk, Poland. Patients were between 27 and 42 weeks of gestation with a singleton pregnancy with symptoms of GH or PE but without co-morbidities. Women with chronic secondary/essential hypertension, immunological diseases like Hashimoto’s disease, diabetes mellitus, pre-existing renal disease, intrauterine fetal death, gestational diabetes, bacteriuria, multiple pregnancies, assisted reproductive technology in pregnancy, and premature rupture of membranes was excluded. Long-term treated patients with aspirin or other anti-inflammatory agents were excluded. Serum samples were taken close to the hospital admission when symptoms of hypertension developed. The patients were hospitalized at various periods of pregnancy. The majority were admitted beyond 34th week of pregnancy. Those admitted prior to 32nd week of pregnancy were admitted due to exacerbation of hypertension, worsening of general condition of FGR. This study was approved by the Bioethics Committee at the Medical University of Gdansk (no. NKBBN/454/2014) and was conducted according to the principles of the Declaration of Helsinki. All participants provided written informed consent to participate in the study.

### Patients

**Figure 1:**
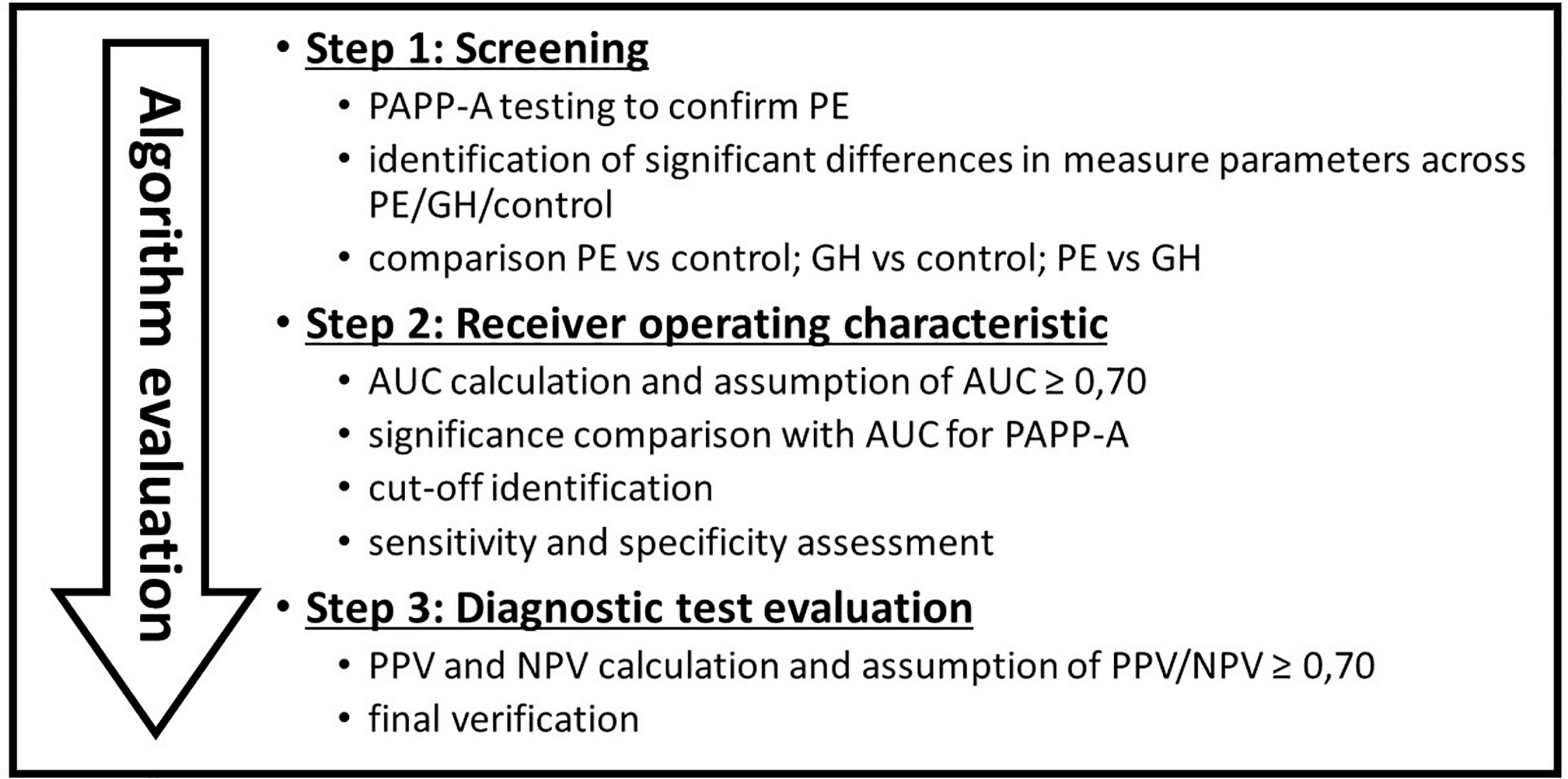
Study flow diagram. Criteria for test selection.

Based on clinical and laboratory evaluations, according to the ISSHP classification, the patients were divided into three groups: the GH group (n = 19) and PE group (n = 28) and the control group (n = 21). The study flow diagram is shown in **Figure 1**, and the baseline characteristics of the study population are provided in **Table 1**.

**Table 1:** Patients’ characteristics.

Patients’ status	PE	GH	Control	p
	n = 44	n = 44	n = 21	
**Age (years, mean ± SD)**	28 ± 4.05	30 ± 4.60	30 ± 3.44	0.323
**Period of gestation**	35	39		
**Body mass index at enrollment (kg/m^2^) (median, min/max)**	30	33	26	<0.001
	(21/46)	(26/42)	(20/34)	
**Parity**				
** 0**	36	34	20	NT
** 1**	7	7	1	NT
** >1**	1	3	0	NT

GH was defined as systolic blood pressure ≥140 mmHg and diastolic blood pressure ≥90 mmHg in a previously normotensive pregnant woman after 20 weeks of gestation without proteinuria or an indication of end-organ dysfunction. PE was diagnosed in patients with high blood pressure (24 h blood pressure records) and new-onset proteinuria, i.e., when resting blood pressure was ≥ 140/90 mmHg on two occasions that were at least 4 h apart, and significant proteinuria was detected in urine samples. Proteinuria was assessed as a urine protein/creatinine ratio (UPCR) ≥30 mg/dl cut-off. In the absence of proteinuria, PE was diagnosed based on hypertension in association with thrombocytopenia (platelet count <150,000/μl), impaired liver function (increased blood levels of liver aminotransferases to twice the normal concentration), a new development of renal insufficiency (elevated serum creatinine >1.02 mg/dl), pulmonary oedema, new-onset of cerebral or visual disturbances, or uteroplacental dysfunction, including FGR. FGR was diagnosed as fetal abdominal circumference/estimated fetal weight <10th percentile combined with pulsatility index in the umbilical artery >95th percentile, or pulsatility index in the uterine artery >95th percentile, or abdominal circumference/estimated fetal weight <3rd percentile, or absent end-diastolic flow in the umbilical artery (22).

### Methods

The patient’s 5 ml whole blood sample was collected to obtain serum. Samples with visible hemolysis or lipemia were rejected and all sera were stored at −70°C before testing. Samples were thawed only once before testing. Cytokines were assessed using 38 Milliplex Multiplex Assays for Luminex (Merck, Germany), and next proteins: Activin A, PAPPA-1, FMS-like tyrosine kinase 3 ligand (FLT-3L), IL-12p40, interferon gamma-induced protein 10 or C-X-C motif chemokine ligand 10 aka CXCL10 (IP-10), monokine induced by gamma interferon or chemokine (C–X–C motif) ligand 9 aka CXCL9 (MIG), monocyte chemoattractant protein 1 or chemokine (C–C motif) ligand 2 aka CCL2 (MCP-1), monocyte chemoattractant protein 3 or chemokine (C–C motif) ligand 7 aka CCL7 (MCP-3), TNF-β were assayed with ELISA (R&D Systems, Inc. Minneapolis, MN). For tested parameters, the manufacturer’s detection limit was: 3.67 pg/ml, 0.053 ng/ml, 0.75 pg/ml, 3.93pg/ml, 2.47 pg/ml, 5.23 pg/ml, 3.33 pg/ml, 8.79 pg/ml, 1.22 pg/ml respectively.

### Statistics

Statistical analyses were done with Statistica (Statsoft, Poland). All variables were expressed as median and range if not normally distributed. Differences between PR, GH, and control groups were tested with a nonparametric Kruskal–Wallis statistical test and multiple comparisons were done with Dunn’s test. For all statistics, the values ≤0.05 were considered significant. For selected parameters, specificity and sensitivity were assessed using ROC analysis, and then negative and positive predictive values were calculated. Heatmaps and cluster analysis were performed in ClustVis (23).

## Results

We have found several markers specific for PE, GH or both and the collected data were given in **Table 2**. For PE *versus* control markers like PAPP-A, activin A, FLT-3L, IL-12p40, IL-22, IP-10, MIG, TNFβ were significant. While for GH *versus* control, FLT-3L, IP-10, MCP-1, MCP-3, MDC/CCL22 ratio were significant. Finally, for PE *versus* GH, activin A, IL-18/IL-2p70 ratio, and IL-2/IL-4 ratio were valid. Each of this was discussed, giving values and range in tested populations. The results were presented according to the study flow diagram (**Figure 1**), first patients were screen with PAPP-A and then using only valid parameters for PE/GH, AUC was calculated to find the markers with high discriminatory capacities (AUC ≥0.7). Finally, diagnostic test evaluation was done and negative/positive predictive values were calculated.

**Table 2:** Screening for PE/GH markers.

Parameter	Kruskal–Wallis test [p]	Dunn’s multiple comparison test (significant if p < 0.05: yes/no)			Parameter	Kruskal–Wallis test [p]	Dunn’s multiple comparison test (significant if p < 0.05: yes/no)		
		PE *vs* GH	PE *vs* control	GH *vs* control			PE *vs* GH	PE *vs* control	GH *vs* control
*PAPP-A	0.006	no	yes	no	*IL-12p40	0.029	no	yes	no
PIGF	0.081	no	no	no	IL-12p70	0.526	no	no	no
*activinA	<0.001	yes	yes	no	*† IL-12p70/40	0.025	no	no	yes
IGFBP-1	0.141	no	no	no	*†IL-18/IL-2p70	0.028	yes	no	no
IGFBP-3	0.087	no	no	no	IL-13	0.348	no	no	no
VEGFR	0.282	no	no	no	IL-15	0.388	no	no	no
sCD40L	0.241	no	no	no	IL-17α	0.980	no	no	no
EGF	0.964	no	no	no	IL-17E	0.514	no	no	no
eotaxin	0.233	no	no	no	IL-17F	0.335	no	no	no
FGF-2	0.576	no	no	no	IL-18	0.105	no	no	no
*FLT-3L	0.008	no	yes	yes	*IL-22	<0.001	no	yes	no
fractalkine	0.816	no	no	no	IL-27	0.056	no	no	no
G-CSF	0.104	no	no	no	*IP-10	<0.001	no	yes	yes
GROα	0.422	no	no	no	*MCP-1	0.009	no	no	yes
IFNα2	0.802	no	no	no	*MCP-3	0.009	no	no	yes
IFNγ	0.762	no	no	no	M-CSF	0.192	no	no	no
IL-1α	0.106	no	no	no	*MDC/CCL22	0.030	no	no	yes
IL-1β	0.553	no	no	no	*MIG	0.002	no	yes	no
IL-1RA	0.586	no	no	no	MIP-1a	0.876	no	no	no
IL-2	0.765	no	no	no	MIP-1b	0.615	no	no	no
IL-3	0.628	no	no	no	PDGF-AA	0.876	no	no	no
IL-4	0.083	no	no	no	PDGF-AB/BB	0.581	no	no	no
IL-5	0.388	no	no	no	TGFα	0.093	no	no	no
IL-6	0.983	no	no	no	TNFα	0.730	no	no	no
IL-7	0.249	no	no	no	*TNFβ	0.012	no	yes	no
IL-8	0.066	no	no	no	VEGFα	0.193	no	no	no
IL-9	0.758	no	no	no	†IFNγ/IL-4	0.750	no	no	no
IL-10	0.230	no	no	no	*†IL-2/IL-4	0.028	yes	no	no

(†) calculated ratio; (*) significant result; significant when p < 0.005.

### General Testing for PE—Correlation With PAPP-A

A comparison with currently-used markers was done. In this study, PAPP-A values were significant when comparing PE *versus* control, but not for GH *versus* control or GH *versus* control. Unfortunately, PIGF values were not significant across tested patients. First, patients were assessed according to the pregnancy-associated plasma protein A (PAPP-A) concentration, a well-defined preeclampsia marker. The lowest values of PAPP-A were found in PE woman reaching 86.57 pg/ml median (min = 75.26; max = 99.28) while for gestational hypertension women (GH), the median was 90.39 pg/ml (min = 74.24; max = 103.0) and for the control group 97.66 pg/ml (min = 66.61; max = 108.2). This was significant (p = 0.006) for PE compared to the control group. The AUC value was 0.76 (95%CI 0.62–0.91; p = 0.001). For the PAPP-A cut-off 90.06 pg/ml sensitivity and specificity was 71.43% (95%CI 47.80–88.72), and 62.96% (95%CI 42.37–80.60) respectively. PAPP-A concentration did not differ in GH (median = 90.39 pg/ml; min = 74.24; max = 103.00) *versus* control or PE significantly.

### Cytokine and Growth Factors Screening for PE

**Figure 2:**
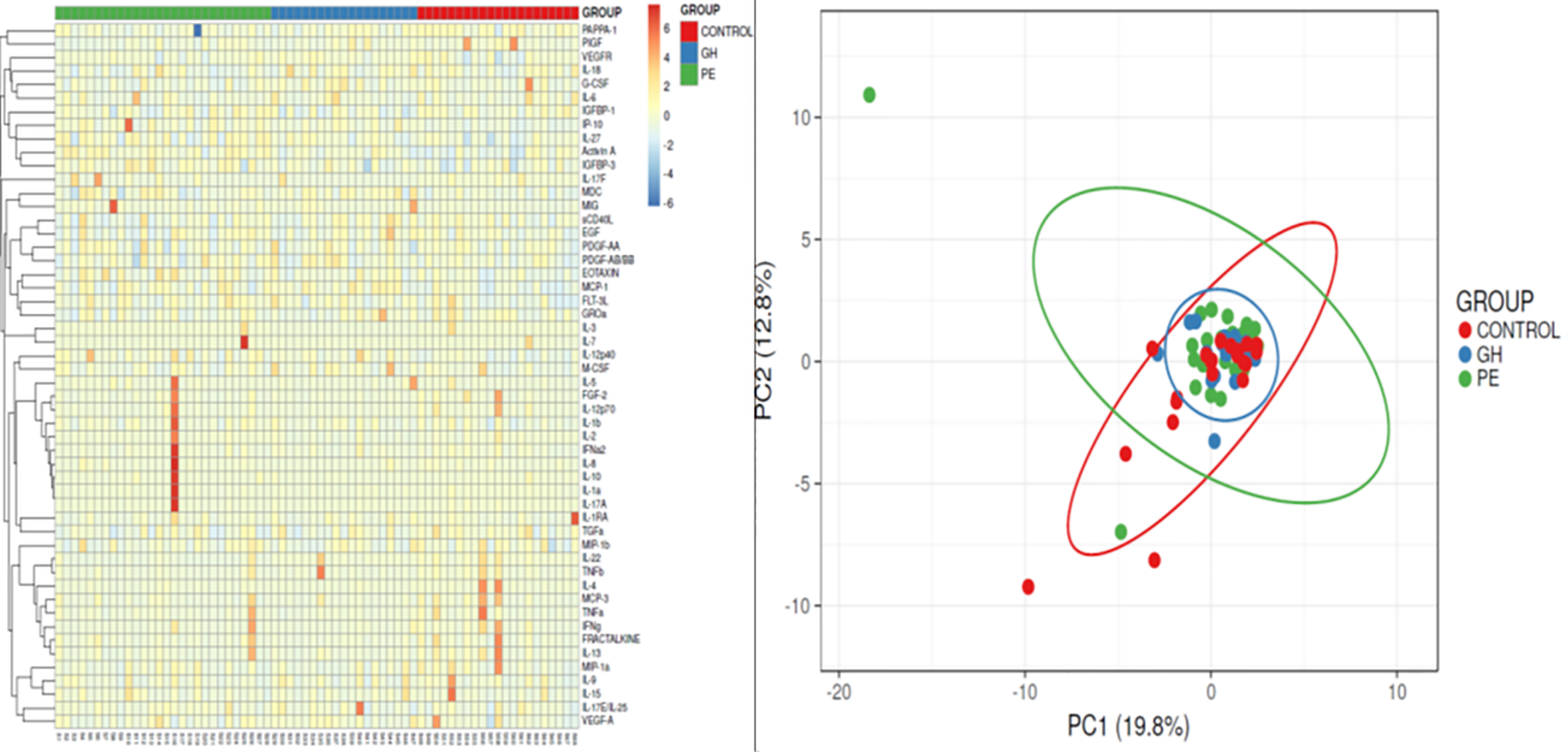
Screening with heatmap and PCA.

Once PE was confirmed by the PAPPA-1, and by basic clinical characteristics, patients were assessed according to the set of 53 cytokines and growth factors. We used the Kruskal–Wallis test, Dunn’s multiple comparison test, heatmap and principal component analysis to screen whether there were any relevant parameters, either for PE or GH, as compared with a healthy pregnant woman. This was summarized in **Table 2** and visualized in **Figure 2**. We have selected eight parameters significant for PE (PAPP-A, activin A, FLT-3L, IL-12p40, IL-22, IP-10, MIG, TNFβ), five for GH (FLT-3L, IP-10, MCP-1, MCP-3, MDC/CCL22 ratio) and three for discriminating between PE *vs* GH patients (activin A, IL-18/IL-2p70 ratio, and IL-2/IL-4 ratio).

### Receiver Operating Characteristics

Next, AUC was calculated and all parameters were above, arbitrarily set, 0.70 cut-off value. Finally, marker’s concentration cut-offs were set to discriminate PE and GH patients with the highest sensitivity and specificity. Finally, parameters with the best characteristics were chosen, as possible markers of preeclampsia. This was summarized in **Table 3**.

**Table 3:** Area under curve (AUC) for selected parameters.

	AUC (95%CI)	p	cut-off [pg/ml]	sensitivity [%] (95%CI)	specificity [%] (95%CI)
**PE *vs* control**					
PAPP-A	0.76 (0.62–0.91)	0.001	>90.06	71.43 (47.80–88.72)	62.96 (42.37–80.60)
activin A	0.83 (0.69–0.96)	<0.001	<1,573.00	73.33 (44.90–92.21)	66.67 (43.03–85.41)
FLT-3L	0.76 (0.61–0.91)	0.003	<28.56	77.78 (52.36–93.59)	69.23 (48.21–85.67)
IL-12p40	0.70 (0.55–0.86)	0.019	<32.99	89.47 (66.86–98.70)	44.44 (25.48–64.67)
IL-22	0.94 (0.87–1.03)	<0.001	>12.47	100 (59.04–100.00)	70.37 (49.82–86.25)
IP-10	0.81 (0.69–0.94)	<0.001	<114.20	84.21 (60.42–96.62)	77.78 (57.74–91.38)
MIG	0.80 (0.67–0.93)	<0.001	<918.30	73.68 (48.80–90.85)	77.78 (57.74–91.38)
TNFβ	0.76 (0.61–0.91)	0.008	>1.99	76.92 (46.19–94.96)	62.96 (42.37–180.60)
**GH *vs* control**					
FLT-3L	0.73 (0.56–0.91)	0.015	<28.99	77.78 (52.36–93.59)	68.42 (43.45–87.42)
†IL-12 p70/p40	0.78 (0.62–0.93)	0.004	>0.06	77.78 (52.36–93.59)	61.11 (35.75–82.70)
IP-10	0.79 (0.65–0.93)	0.002	<118.20	84.21 (60.42–96.62)	61.11 (35.75–82.70)
MCP-1	0.80 (0.65–0.94)	0.002	<360.60	84.21 (60.42–96.62)	63.16 (38.36–83.71)
MCP-3	0.80 (0.65–0.94)	0.002	<360.60	84.21 (60.42–96.62)	63.16 (38.36–83.71)
MDC/CCL22	0.74 (0.58–0.91)	0.010	<492.40	84.21 (60.42–96.62)	68.42 (43.45–87.42)
**PE *vs* GH**					
activin A	0.77 (0.60–0.93)	0.007	<1,727.00	86.67 (59.54–98.34)	61.90 (38.44–81.89)
†IL-18/12p70	0.77 (0.63–0.91)	0.002	>21.73	77.78 (52.36–93.59)	65.38 (44.33–82.79)
† IL-2/IL-4	0.76 (0.63–0.90)	0.002	>21.73	77.78 (52.36–93.59)	65.38 (44.33–82.79)

(†) calculated ratio; significant when p < 0.005.

The serum IL-22 was significant (p <0.001) for PE compared with control group, as the median was 12.32 pg/ml (95%CI 12.87–26.72) *vs* 93.29 pg/ml (95%CI 44.52–234.30). In GH patients, IL-22 was 20.90 pg/ml (95%CI 13.34–29.86), and did not differ from PE. The calculated AUC for PE *vs* control was 0.94 (95%CI 0.87–1.03; p <0.001), and sensitivity and specificity for the level of 12.47 pg/ml was 100% (95%CI 59.04–100.00) and 70.37% (95%CI 49.82–86.25) respectively.

Activin A was noted as characteristic for preeclampsia, as median value was 1,968 pg/ml (95%CI 1,609.00–2,068.00) for PE, 1,392.00 pg/ml (95%CI 1,090.00–1,604.00) for hypertensive pregnancies, and 986.40 pg/ml (95%CI 858.30–1,440.00) for control. This was significant (p = 0.001) in PE *vs* GH and control. AUC for PE *vs* control comparison was 0.83 (95%CI 0.69–0.96; p <0.001), and for 1,535 pg/ml cut-off sensitivity was 73.33% (95%CI 44.90–92.21), and specificity 66.67% (95%CI 43.03–85.21). For PE *vs* GH comparison, AUC was 0.77 (95%CI 0.60–0.93; p <0.007), and for 1,727 pg/ml cut-off sensitivity was 86.67% (95%CI 59.54–98.34), and specificity 61.90% (95%CI 38.44–81.89).

There was an increase in FLT-3L concentration in both PE and GH woman compared to the control group (p = 0.008), 33.83 pg/ml (95%CI 29.02–39.91) and 30.46 pg/ml (95%CI 25.7–39.13) *vs* 19.64 pg/ml (95%CI 16.31–29.28). But, FLT-3L did not differ between PE and GH. The AUC for PE *vs* control was 0.76 (95%CI 0.61–0.91; p = 0.003). For the 28.56 pg/ml cut-off value sensitivity and specificity was 77.78% (95%CI 52.36–93.59), and 69.23% (95%CI 48.21–85.67).

Next the Th1/Th2 milieu was evaluated by the mean of IL-12 and IL-18, its key-regulators, and IL-2, IL-4 and IFNγ. The IL-12p40 serum concentration was increased, as significantly (p = 0.030) higher levels in PE *vs* control were noted. Median IL-12p40 concentration was 27.88 pg/ml (95%CI 27.04–40.51) in PE, 28.61 pg/ml (95%CI 24.87–35.21) in GH, and 20.88 pg/ml (95%CI 17.59–26.88) in control group. The AUC, for the PE *vs* control, was 0.70 (95%CI 0.55–0.86), and p was 0.019. The maximum 89.47% (95%CI 66.86–98.70%) sensitivity, and 44.44% (95%CI 25.48–64.67) specificity was for the 32.99 pg/ml IL-12p40 serum concentration. Once IL-12p70/p40 ratio was calculated, both median values for PE (0.06; 95%CI 0.05–0.14) and GH (0.06; 95%CI 0.05–0.07) were lower as compared with control (0.9; 95%CI 0.08–0.40), but only significant for a GH *vs* control comparison (p = 0.026). Next, IL-18/IL-12p70 ratio was calculated, and median for PE and GH was 15.25 (95%CI 12.04–27.56) and 30.91 (95%CI 24.19–42.97), compared to control, 21.05 (95%CI 15.26–34.92). This was significant, as PE patients differ from GH patients (p = 0.022).The AUC for PE *vs* GH was 0.77 (95%CI 0.63–0.91; p = 0.002), and the 77.78% (95%CI 52.36–93.59%) sensitivity, and 65.38% (95%CI 44.33–82.79) specificity was for the 21.73 ratio was estimated.

The level of IP-10 differs both in PE and GH from control (p = 0.001). The median 144.9 (95%CI 124.5–180.0) and 126.7 (95%CI 110.2–154.9) IP-10 values were noted in PE and GH, which contrast with 82.39 pg/ml (95%CI 67.3–101.2) median for control group. The AUC for PE *vs* control was 0.81 (95%CI 0.69–0.94), and p was <0.001. The 114.20 pg/ml IP-10 cut-off was characteristic for 84.21% (95%CI 60.42–96.62) sensitivity, and 77.78% (95%CI 57.74–91.38) specificity. What’s more, there was a correlation in PE woman between IP-10 *vs* FLT-3L (p = 0.014; Spearman r was 0.483) and IP-10 vs IFNγ (p = 0.013; Spearman r was 0.497). For the GH and control AUC was 0.79 (95%CI 0.65–0.93; p = 0.002) and for the 118.20 pg/ml cut-of specificity and sensitivity was 84.21% (95%CI 60.42–96.62) and 61.11 (35.75–82.70) respectively.

Another marker was CXCL9/MIG increased concentration in PE (p = 0.002), as there was a higher concentration of CXCL9/MIG in PE vs control but not GH. For PE, median CXCL9/MIG was 1,117.00 pg/ml (95%CI 1,066.00–1,521.00) in PE, 747.60 pg/ml (95%CI 879.70–1,481.00) in GH, and 790.00 pg/ml (95%CI 650.10–900.50) in control, respectively. The AUC value was 0.80 (95%CI 0.67–0.93), and p value was <0.001. For the 918.30 pg/ml cut off value sensitivity and specificity was 73.68% (95%CI 48.80–90.85), and 77.78% (95%CI 57.74–91.38). Finally, CXCL9/MIG was not correlated with IL-17A, as p was 0.115 and Spearman r was −0.316.

In PE group the TNFβ was significantly (p = 0.013) reduced, as compared with control, but did not differ from GH patients. The median TNFβ for control was 3.98 pg/ml (95%CI 3.06–31.24) and for PE and GH 1.02 pg/ml (95%CI 1.87–7.90) and 1.00 pg/ml (95%CI 0.41–5.67).

Finally, MCP-1, MCP-3, and MDC were increased in GH patients, as compared with PE and control (p = 0.009; p = 0.009; p = 0.031). The median level of MCP-1, MCP-3, and MDC in GH *vs* control was: 409.3 pg/ml (95%CI 351.4–467.2) *vs* 274.0 pg/m (95%CI 242.2–332.1); 406.5 pg/ml (95%CI 351.4–467.2) *vs* 274.0 pg/ml (95%CI 242.2–332.1); and 548.6 pg/m (95%CI 474.6–620.8) *vs* 426.2 pg/m (95%CI 377.4–476.9) respectively. Thus, AUC was 0.80 (95%CI 0.65–0.94; p = 0.002); 0.80 (95%CI 0.65–0.4; p = 0.002); and 0.74 (95%CI 0.58–0.91; p = 0.010). The highest sensitivity and specificity was given for 360.60 pg/ml MCP-1 and MCP-3 cut-offs and 492.40 pg/ml MDC cut-off.

We have then checked whether AUC for IL-22 and MIG significantly differed from the PAPP-A AUC value. This was true for PAPP-A *vs* IL-22 as p was 0.030 but not for PAPP-A vs MIG, as p was 0.712.

### Diagnostic Test Evaluation

**Figure 3:**
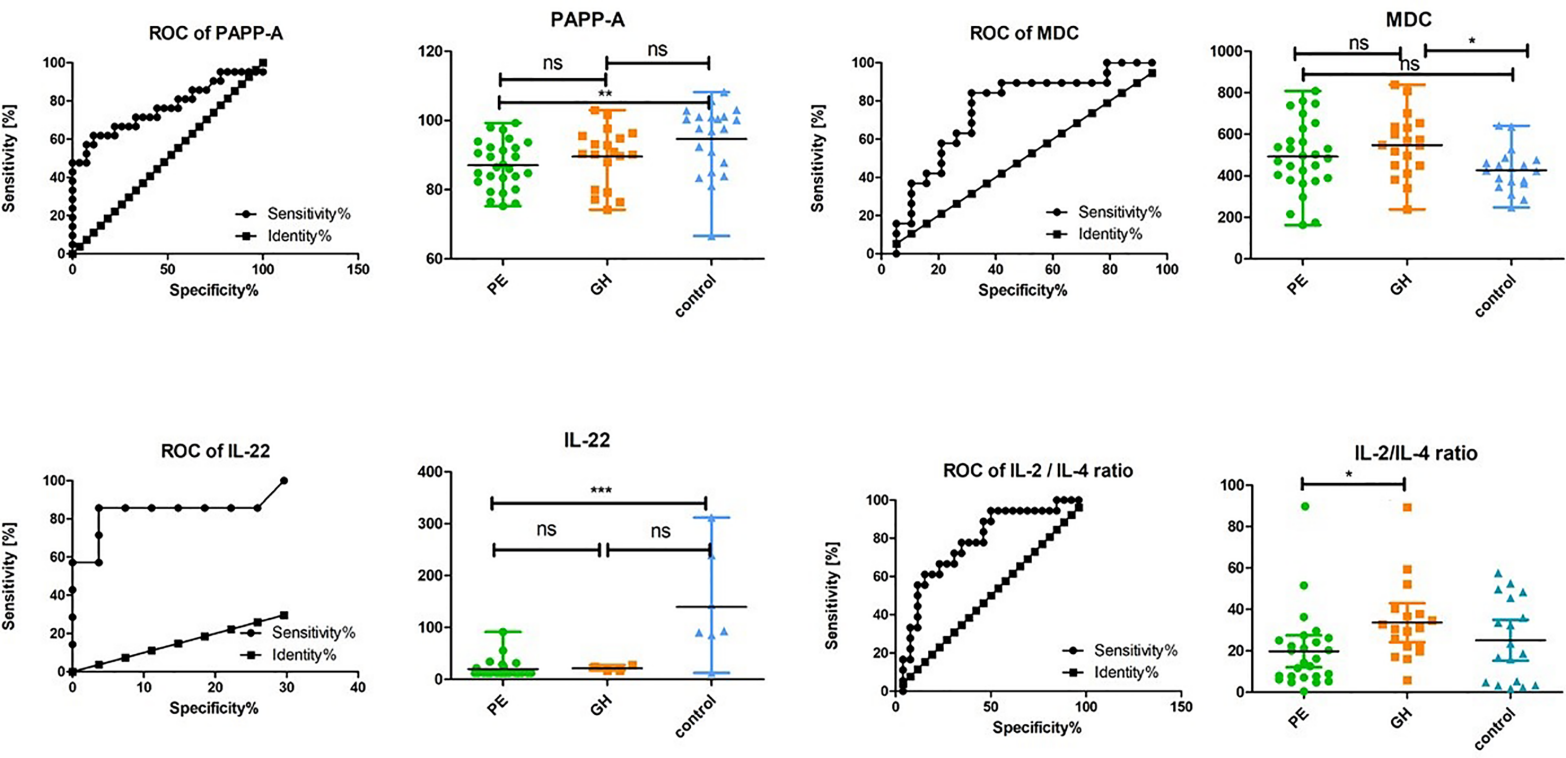
AUC of selected parameters. *p < 0,050; **p < 0,025; ***p < 0,001; ns, non-significant.

Finally, for IL-22, MCP-1 or MCP-3, MDC, L-18/12p70 ratio and IL-2/IL-4 ratio positive and negative predictive values (PPV, NPV) were calculated and summarized in **Table 4** and visualized in **Figure 3**. The best results were obtained for IL-22, MDC, and IL-2/IL-4 ratio, with PPV above 80% for PE *vs* control and GH vs control, and IL-2/IL-4 ratio NPV above 80% for PE *vs* GH comparison. In this way, the preeclampsia diagnostic algorithm can be proposed.

**Table 4:** Diagnostic test evaluation.

	PPV [%] (95%CI)	NPV [%] (95%CI)
**PE *vs* control**		
IL-22	100.00	100.00
**GH *vs* control**		
MCP-1 or MCP-3	80.00 (57.26–92.27)	69.7 (55.15–80.95)
MDC/CCL22	81.25 (59.48–92.75)	72.73 (57.22–84.17)
**PE *vs* GH**		
†IL-18/12p70	60.87 (46.47–73.59)	80.95 (63.15–91.34)
† IL-2/IL-4	60.87 (46.47–73.59)	80.95 (63.15–91.34)

(†) calculated ratio.

## Discussion

In this research, we have extensively studied a wide range of serum proteins, mainly cytokines, chemokines and growth factors, as potential markers of preeclampsia. We have identified three parameters from over fifty proteins, with a potential significance in the diagnosis of PE. The in-depth analysis showed that the IL-22, MDC, and IL2/IL-4 ratio can be used to discriminate between preeclampsia, gestation hypertension and healthy pregnancy. Finally, we have proposed a diagnostic algorithm with well-defined cut-offs with potential clinical usefulness. What’s more, the indicated parameters have been assessed with AUC, NPV, and PPV showing strong diagnostic capacities in screening for PE.

It should be noted that even routinely used diagnostic parameters like PAPP-A, PIGF, and FLT-3 have not been definitive for PE. This was because some of them provide the best specificity and sensitivity when tested in a fixed time. For example, excellent concordance of PAPP-A concentration with PE was noted in the first trimester. There is also an approach to use both laboratory parameters and clinical observations, like Doppler ultrasonography, to predict PE (24). For this reason searching for novel preeclampsia markers is highly justified and using a combined parameters approach is effective. Another importance of this work is that we have selected a common set of cytokines, that can be easily assayed with ELISA or other immunological methods, and some of them like IL-2 and IL-4 can be tested with high throughput laboratory analyzers. What’s more, we have recruited women between the second and third trimesters of pregnancy when the symptoms of hypertension are common. This could be another advantage, as there is no need to screen patients in a fixed period of gestation.

In this study decreased levels of IL-22 have been shown as specific for PE with PPV reaching 100%. IL-22 is a proinflammatory cytokine, of Th1 origin, important for acute phase proteins production. According to the recent meta-analysis, there are contradictory data on IL-22 in PE (25). One possible explanation for that is, that in one study severe PE woman were recruited (26). In contrast, we have recruited patients shortly after symptoms occurred, and no evidence for general inflammation was noted, as tested by IL-1, IL-6, IL-17, and TNFα in PE compared to GH and control. Another was that we have observed a general shift toward Th2 cytokines as indicated by the IL-2/IL-4 ratio and IFNγ/IL-4 ratio. The Th1/Th2 balance was essential to discriminate between PE and GH. NPV for both IL-2/IL-4 ratio and IL-18/12p70 ratio was above 80%, which means that the negative result (below the cut-off) was specific for GH. Here we have proposed to use the IL-2/IL-4 ratio in our algorithm, as these parameters are available for several analytical platforms.

MDC, aka CCL22, is a macrophage-derived chemokine that attracts Th2 lymphocytes. Several immune cells secrete MDC upon stimulation. The only known receptor for MDC is CCR4, a receptor constitutively expressed by the regulatory T cells (Treg) and Th2 lymphocytes upon activation. While for activated T lymphocytes CCR4/CCL22 plays a role in allergy development, this pathway is essential for Tregs migration (27) One of the examples of this axis can be found in diabetes type 1, where CCL22-dependent Treg migration to the pancreatic islets may downregulate inflammation and disease progression (28). For these reasons, MDC is an important immune checkpoint, and in our study increased levels were found in GH patients. These findings can be also important for future hypertension drugs development.

This paper also reveals some new information on the immunopathogenesis of preeclampsia, such as the increased level of FLT-3L (fms-like tyrosine kinase 3 ligands) in PE woman. We have noted that FLT-3L serum level in PE and GH patients was important when compared to the control. The FLT-3 is a tyrosine kinase receptor, considered to be involved in the early hematopoiesis, and which plays a role in dendritic cell mobilization and development, as it’s an essential signal for plasmacytoid dendritic cells growth (29). Yet, several mutations in the FLT-3 gene have been identified in acute leukemias, and serum FLT-3L was shown to be a useful marker in the classification of acute myeloid leukemia (30). There are very limited data on FLT-3 or FLT-3L in the pathology of hypertension, more so in preeclampsia development, but very recent experiments using an animal model indicated that FLT-3L mediated dendritic cells activation leading to oxidative stress, fluids retention, and finally blood pressure increase (31). No variation was found between GH and PE woman in our data, which probably shows that FLT-3L is specific for a general mechanism of hypertension, rather than for PE only. Importantly FLT-3L was correlated with IP-10, an essential pro-inflammatory chemokine, regulating chemotaxis and endothelial adhesion of T lymphocytes and NK cells. IP-10 also has anti-angiogenic capacities, and increased concentration in PE was demonstrated (32). We have noted that IP-10 was positively correlated with IFNγ, a significant Th1 cytokine.

Further, IL-12p40 concentration was higher in PE but not in GH woman. This must be discussed, as the IL-12 (a protein about 70 kDA) is composed of two subunits, IL-12p40 (40 kDA) and IL-12p35 (35 kDA). In this study, IL-12p70 was proportional across all participants, but not IL-12p40. Importantly, free IL-12p40 has an antagonistic effect on IL-12p70, as it blocks receptor binding. Therefore, the biological effect of IL-12p70, like the proliferation of T cells toward Th1 phenotype, is diminished (33). IL-12 serves as a link between innate and adaptive immunity, as it’s produced by dendritic cells or macrophages and stimulates T cells to secrete IFNγ essential Th1 factor. Moreover, IL-12p40 is a subunit of another IL-12 family member, IL-23. The IL-23 drives CD4 T lymphocytes towards Th17 phenotype, a potent pro-inflammatory cell. Hence, IL-12p40 may contribute conversely to the inflammation, depending on the IL-12/IL-23 pathway (33). Interestingly, IL-12p40 was increased in older individuals, and in correlation with decreased CD3 T lymphocytes, mainly CD4+. Collectively, this data supports previous observations that preeclampsia was characterized by the cytokine imbalance toward Th1 phenotype (34). What’s more IL-12 and IL-18 inhibit angiogenesis *via* the induction of IFN-γ, which in turn induces the production of CXCL9, 10 and 11, thus human CXCL9 is a potent inhibitor of angiogenesis (35).

According to our findings, preeclampsia was associated with an overall pro-inflammatory systemic environment. Elevated pro-inflammatory cytokines, chemokines and adhesion molecules in the maternal circulation may play a central role in the excessive systemic inflammatory response, as well as in the generalized endothelial dysfunction characteristics of the maternal syndrome of preeclampsia (15).

There were some limitations in this study, like inability for strict risk stratification, that in our opinion require to plan the new experiment. Another was that only two commonly-used markers of PE were used to compare our findings. Before application to the clinics, the algorithm should be checked in different patients’ cohorts to address some important questions, like impact of parity, comorbidities, or patient’s age on test results. Also, in depth validation with other PE markers is needed.

In conclusion, we have demonstrated that a set of cytokines, IL-22, MDC, and IL-2/IL-4 can be used to diagnose preeclampsia, and what’s more to discriminate from gestational hypertension. According to our data, the algorithm is adequate and enables correct identification of PE and cross-diagnosis of gestation hypertension. Once adopted for the clinical setting, it could help in decision-making on the risk of PE development and a need for straight patient monitoring. Interesting, some of the investigated markers were closely associated with the CD4+ T lymphocytes (Th1/Th2 balance), and this data may influence future development of cellular therapies, to overcame or slow down the disease development. One important favor of this algorithm was analytics as discussed previously, with use of well-established methodology (ELISA based assays) and small amount of patients serum for testing. We believe that our finding will result in better patient management and risk stratification, the aimed characteristics of personalized medicine.

## Data Availability Statement

The raw data supporting the conclusions of this article will be made available by the authors, without undue reservation.

## Ethics Statement

The studies involving human participants were reviewed and approved by the Bioethics Committee at the Medical University of Gdansk (no. NKBBN/454/2014). The patients/participants provided their written informed consent to participate in this study.

## Author Contributions

Conceptualization: KS, N-MT, and PT. Methodology: MZ. Software: MZ. Validation: KS and MZ. Formal analysis: KS, N-MT, and MZ. Investigation: JJ-B and KS. Resources: PA, DZ, MJ, KL, RS-S, KaP, KrP, and JB. Data curation: KaP, DZ, MJ, KrP, and KS. Writing—original draft preparation: KS, MZ, DZ, and N-MT. Writing—review and editing: KS, MZ, and DZ. Visualization: MZ. Supervision: KP, PT, N-MT, KS, and SK. Project administration: KS, MZ, and N-MT. Funding acquisition: KS, N-MT, and PT. All authors contributed to the article and approved the submitted version.

## Funding

This study was supported with funds from the Polish National Science Center based on Decision no. 2014/15/B/NZ5/03499.

## Conflict of Interest

The authors declare that the research was conducted in the absence of any commercial or financial relationships that could be construed as a potential conflict of interest.
